# Aldose Reductase: An Emerging Target for Development of Interventions for Diabetic Cardiovascular Complications

**DOI:** 10.3389/fendo.2021.636267

**Published:** 2021-03-11

**Authors:** Sravya Jannapureddy, Mira Sharma, Gautham Yepuri, Ann Marie Schmidt, Ravichandran Ramasamy

**Affiliations:** Diabetes Research Program, Division of Endocrinology, Diabetes and Metabolism, Department of Medicine, NYU Grossman School of Medicine, New York, NY, United States

**Keywords:** diabetes, cardiovascular diabetic complications, aldose reductase, polyol pathway, hyperglycemia, aldose reductase inhibitor, cardiovascular disease

## Abstract

Diabetes is a leading cause of cardiovascular morbidity and mortality. Despite numerous treatments for cardiovascular disease (CVD), for patients with diabetes, these therapies provide less benefit for protection from CVD. These considerations spur the concept that diabetes-specific, disease-modifying therapies are essential to identify especially as the diabetes epidemic continues to expand. In this context, high levels of blood glucose stimulate the flux *via* aldose reductase (AR) pathway leading to metabolic and signaling changes in cells of the cardiovascular system. In animal models flux *via* AR in hearts is increased by diabetes and ischemia and its inhibition protects diabetic and non-diabetic hearts from ischemia-reperfusion injury. In mouse models of diabetic atherosclerosis, human AR expression accelerates progression and impairs regression of atherosclerotic plaques. Genetic studies have revealed that single nucleotide polymorphisms (SNPs) of the *ALD2* (*human AR gene*) is associated with diabetic complications, including cardiorenal complications. This Review presents current knowledge regarding the roles for AR in the causes and consequences of diabetic cardiovascular disease and the status of AR inhibitors in clinical trials. Studies from both human subjects and animal models are presented to highlight the breadth of evidence linking AR to the cardiovascular consequences of diabetes.

## Introduction

Diabetes prevalence worldwide has been increasing at an alarming rate. World Health organization estimates that currently greater than 400 million people live with diabetes (https://www.who.int/health-topics/diabetes). As the number of people with diabetes has increased, consequent increases in diabetic complications has been observed (1, 2). Among the various diabetic complications, cardiovascular disease (CVD) is the leading cause of morbidity and mortality in patients with diabetes mellitus (3). CVD entities include increased sensitivity of diabetic myocardium to ischemic episodes (4) and diabetic cardiomyopathy, manifested as a subnormal functional response of the diabetic heart independent of coronary artery disease (5, 6). Macrovascular disease in patients with diabetes, includes, atherosclerosis (7), coronary artery disease (CAD), peripheral vascular disease (PVD) and stroke (8), and restenosis of large vessels (9–11). The United Kingdom Prospective Diabetes Study (UKPDS) demonstrated that despite significant reductions in HbA_1c_, diabetes related mortality and myocardial infarction (MI) events were not reduced (12).

CVD significantly reduces the median life expectancy for diabetic adults in the 55–64 age group (13, 14). This is likely due to diabetes specific cardiovascular disorders. Key among them is the accelerated atherosclerosis in diabetes, with greater infiltration of inflammatory cells, and larger necrotic core size (15). While the deaths due to CAD have declined in the general population, the reduction in deaths due to CAD has been much less dramatic in diabetic patients (16). Another factor contributing to CV death in diabetics is heart failure. Prevalence of diastolic heart failure with preserved ejection fraction (HFpEF) and systolic heart failure with reduced ejection fraction (HFrEF) are higher in patients with diabetes compared to those without diabetes. While the precise mechanisms by which diabetes mediates heart failure are unknown, contributors include impaired endothelial dysfunction, pathways driving fibrosis, cardiomyocyte dysfunction, and defective remodeling after myocardial infarction (17, 18). Similarly, the Framingham Heart Study showed that diabetes independently increases the risk of heart failure (19–21). Recently meta-analysis of sixteen CV outcome trials by Sacre et al. (22) found that hospitalization for heart failure and myocardial infarction are the most frequent CV events in clinical trials in Type 2 diabetes.

High rate of mortality, post-MI, has been observed in people with diabetes vs those without diabetes (21, 23, 24), presumably due to ventricular arrhythmia (25). Mechanisms causing arrhythmias in diabetes include calcium channel function changes driven by downregulation of SERCA2a and increased phosphorylation of the ryanodine receptor (26, 27), oxidative stress (28, 29), AGEs-RAGE axis (29–31). Cardiac autonomic neuropathy prevalent in patients with diabetes, has been linked to increased risk for fatal cardiac arrhythmias (32).

In the recent outbreak of the coronavirus disease 2019 (COVID-19), diabetes and cardiovascular disease are risk factors for severe adverse clinical outcome in COVID19 patients (33). Emerging data reveal that diabetes and obesity are among the strong predictors for hospitalization among COVID-19 patients and risk factor for severe COVID-19 morbidity and mortality (34–38). In these hospitalized COVID-19 patients, myocardial infarction with or without obstructive coronary lesions (33, 39) were observed. These and the above findings strongly highlight the urgent need for focused therapies for alleviating the devastating impact of cardiovascular complications induced by diabetes. Global efforts are underway to find more effective strategies to mitigate and or attenuate the devastating consequences of diabetic cardiovascular complications.

In this review, we will focus on aldose reductase (AR), its possible link to the cardiovascular complications of diabetes mellitus and the potential impact of pharmacological inhibition of AR on cardiovascular complications of diabetes.

## Hyperglycemia in Cardiovascular Cells

One of the key mechanisms by which chronic hyperglycemia(CH) exerts its deleterious effects on CV tissue involves nonenzymatic glycation reactions of reducing sugars with free amino groups of proteins, DNA, and lipids. Amadori products formed by this reaction leads to the formation of advanced glycation end products (AGEs) (40–48). These derivatives can bind to pre-existing cell surface receptors of AGEs and such interactions often lead to generation of reactive oxygen species through perturbation of NADPH oxidase (33–38, 49). CV tissue is less dependent on insulin for glucose uptake from extracellular environment due to abundance of GLUT1, an insulin independent glucose transporter in the plasma membrane (50, 51). During CH, there is chronic and abnormal influx of extracellular glucose due to down regulation of GLUTs altering the biochemical homeostasis of cardiovascular cells (52, 53). Consequently, changes, in flux *via* the polyol pathway, cytoplasmic redox state, activity of specific isoforms of protein kinase C, in the glucosamine biosynthesis pathway, and production of glycating species are observed (9, 53–61).

The present review mainly focuses on aldose reductase (AR), the first enzyme of the polyol pathway that regulates the uptake of excess glucose by the cardiovascular cells.

## Properties of AR and Its Gene, *ALD2*

Aldose reductase (E.C. 1.1.1.21; AKR1B1, ALD2, or AR), a monomeric enzyme of ~35,900 Daltons, belongs to the aldo-keto reductase superfamily (62–67). The enzyme reversibly binds NADPH when it reduces an aldehydic substrate to the corresponding alcohol, e.g., glucose to sorbitol.

AR reduces a variety of aldehydic substrates with differing affinities (68, 69). The enzyme efficiently catalyzes reduction of glyceraldehyde, 4-hydorxynonenal (4-HNE), 2-methylpentanal, methylglyoxal, retinoids and host of other aldehydes (69–71). These AR studies determined that the *K_m_* values for the above substrates are in the range of 8 to 50 µmol/L. For glucose, Inagaki et al. (72) and Grimshaw (73) showed for AR a K_m_ for the open chain of glucose of 0.66 µmol/L.

Oxidation of cysteine residue, Cys 298 causes AR to exhibit altered activity and inhibitor sensitivity (74). AR activity is altered by S−nitrosothiols (75), activated by nitric oxide (NO) under ischemic/acidic conditions (76) or inhibited by elevated NO levels in non-acidic conditions (77). In human tissues AR occurs mostly in the reduced enzyme form (78).

The human AR gene (*ALD2* or *AKR1B1*), approximately 18 kilobases (kb) long and includes ten exons coding for 316 amino acids, has been mapped to locus q35 on human chromosome 7 (79, 80). The TATA box (at -37), a CCAAT box (−104), and an androgen-like response element (−396–382) are in the *ALD2* promoter region of (81). The region containing three osmotic response elements: OreA, OreB and OreC reside upstream of the transcription start site (82).

*ALD2* gene polymorphisms have been found to be associated with most diabetic complications (83, 84). Microsatellite polymorphism in (AC)_n_ repeat region located ~2.1 kb upstream of the transcription start site was first identified in patients with diabetic retinopathy (85). Subsequent studies detected single nucleotide polymorphisms C(-106)T (86) and C(−12)G (87) in the basal promoter region of the *ALD2* gene. An intragenic polymorphism in the BamHI site consisting of an A to C substitution associated with diabetic retinopathy, was also identified (88). Studies by Demaine et al. and Moczulski et al. (89, 90) showed that the (AC)_n_ and C(-106)T polymorphisms are closely linked. Majority of studies have demonstrated an association between polymorphisms in the *ALD2* gene and the increased risk for rapid onset or increased prevalence of diabetic complications. The “Z-2” (AC)_23_ microsatellite polymorphism has been associated with high expression levels of AR (91) and with diabetic retinopathy (85, 89), diabetic nephropathy (90–93). The link between Z-2 allele and diabetic neuropathy is rather modest (94).

In some studies an association between *ALD2* alleles and complications risk has not been detected (95, 96). In one study of Type 2 diabetic patients, although no association of Z−2 with proteinuria was found, a statistically significant association of erythrocyte AR concentration with proteinuria was found (97). *ALD2* gene polymorphism has been detected in Type 2 diabetic patients with cardiorenal complications (98) and microangiopathy (99). It is important to note that most studies across the globe has demonstrated link between diabetic complications and *AL2* polymorphisms (100–104).

## Polyol Pathway and the Osmotic Hypothesis

Based on thee replicated genetic links, the impact of chronically elevated glucose metabolism *via* the AR pathway aka polyol pathway has received considerable attention in the study of diabetic complications. In this pathway (105), AR in the presence of NADPH reduces glucose to sorbitol, while sorbitol dehydrogenase (SDH) uses NAD^+^ to oxidize sorbitol to fructose. The pioneering studies of Kinoshita, Gabbay, Dvornik and colleagues (106) demonstrated the presence of elevated polyol pathway intermediates in diabetic rat tissues and suggested a pathogenic link to diabetic complications. In the seminal “Osmotic Hypothesis” paradigm, high levels of glucose are metabolized through AR and SDH to sorbitol and fructose. Accumulation of sorbitol in tissues like eye lens induces a osmosis driven cascade of altered ion and metabolite homeostasis, culminating in the formation of the sugar cataract (106). Data demonstrating an accelerated rate of sorbitol accumulation and cataract formation in human AR transgenic, and SDH-deficient mice (107) provides clear confirmation of this mechanism for sugar cataract formation.

## Polyol Pathway and Metabolic Flux Hypothesis

The past several decades of research have reemphasized that in many tissues/cells the polyol pathway is integrally linked *via* its coenzymes to various metabolic and signaling pathways (108–111). In studies involving the lens tissue, it was shown that increased flux *via* the polyol pathway, increased turnover of NADPH (112) and that AR and antioxidant defense enzyme glutathione reductase compete for the same pool of cytoplasmic NADPH. Another study showed that increased metabolic flux *via* the polyol pathway impairs the glycolysis in diabetic hearts, resulting from competition between SDH and glyceraldehyde-3-phosphate dehydrogenase (GAPDH) for cytosolic NAD^+^ (58). Furthermore, studies by Williamson and his team have demonstrated increased polyol pathway flux modulates the ratio of free cytosolic NADH to NAD^+^ and consequently impairs neural and vascular function (113–117).

Realization that in conjunction with possible osmotic stress in vascular tissue, excess metabolic flux of glucose through AR affects key pathways linked to diabetic complications *via* its ability to generate precursors/intermediates/activators, has heightened interest in AR and the polyol pathway.

The presence and levels of AR vary in tissues and cells (118), with the inner medulla of kidney expressing the highest amount of AR (119). While sciatic nerve, lens, testis, heart, and cornea, express high levels of AR, organs/tissues such as liver, renal cortex, stomach, spleen, lung, small intestine, and colon express low levels of AR (119). AR is present in cells such as cardiomyocytes, endothelial cells, smooth muscle cells, and fibroblasts. In this review, we summarize the key data on AR and evidence linking AR to cardiovascular complications in diabetes.

## AR and Its Physiological Role

To date, the basic physiological function of AR remains elusive (120). By synthesizing intracellular sorbitol, AR forms parts of a multi-tiered renal osmolyte system that helps protect cells in the renal inner medulla from the locally high osmotic stress (121, 122). Interestingly, AR inhibiton results in upregulation of sorbitol compensatory pathways in the renal osmolyte system (123).

Several potential physiological roles have been proposed, they include (a) generation of intermediates to facilitate production of advanced glycation end product precursors (124–127), (b) process to divert glucose from glycolysis and glucose oxidation (53), (c) participation in the metabolism of steroids (128), norepinephrine intermediates (129), detoxification of aldehydes, e.g., (130), or of their glutathionylated derivatives (131). Like AR, aldehyde dehydrogenase 2 (ALDH2) has been shown to detoxify 4-hydroxynonenal (4-HNE) and is expressed in cardiovascular cells (132, 133). Furthermore, studies have shown that 4HNE is a substrate ALDH2, with *K*_m_ and *V*_max_ values of 14.3 μM and 3.5 nmol min^-1^ mg protein^-1^, respectively (134, 135). The fact that AR, aldehyde reductases, and aldehyde dehydrogenases can essentially compete for various aldehydes (136, 137), makes it challenging to determine specific physiological role for AR.

**Figure 1:**
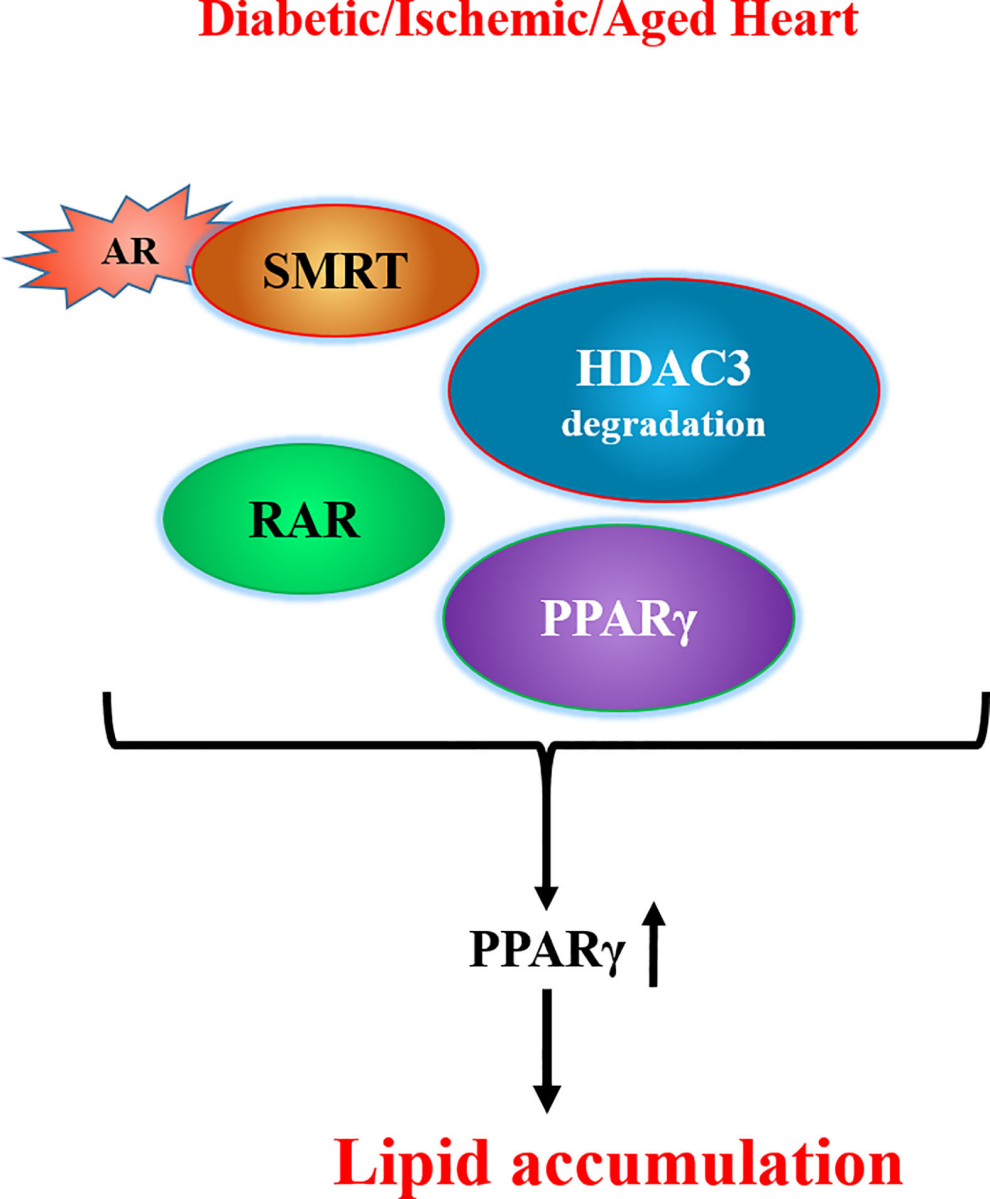
Scheme showing competition between AR and HDAC3 for the DAD of SMRT/NCOR1 and consequent transcriptional changes leading to lipid accumulation. [adapted from (128)]. AR denotes aldose reductase; DAD refers to deacetylation domain of the nuclear corepressors, SMRT refers to silencing mediator of retinoic and thyroid receptor, NCOR1 denotes nuclear corepressor 1, RAR denotes retinoic acid receptor, HDAC3 denotes histone deacetylase 3.

In addition to the enzymatic activity, two recent studies have revealed other functions for AR. First, our studies (138) showed that the interaction of AR with deacetylation domain (DAD) of the nuclear corepressors, silencing mediator of retinoic and thyroid receptor (SMRT) and nuclear corepressor 1 (NCOR1), could lead to histone deacetylase 3 (HDAC3) degradation (**Figure 1**). HDAC3 binds to the DAD of either (SMRT) or (NCOR1) and protects itself from degradation (139). We observed that interaction of AR with DAD of SMRT/NCOR1 in hearts of ischemic, diabetic, and aged mice drives HDAC3 degradation, consequently leading to PPARγ activation and lipid accumulation in the hearts  (138). These findings revealed a novel role for AR in modulating lipid metabolism *via* its ability to regulate HDAC3 degradation and consequent activation of PPARγ.

Second, AR actions independent of its enzymatic activity were revealed in a study by Shimizu et al. (140). Using phosphoproteome analysis and molecular studies, they showed that AR phosphorylation/dephosphorylation is essential for the transduction of T cell receptor-mediated T-cell stimulatory signals. Notably, they showed that AR expression in T cells was unaffected by TCR stimulation or by the presence of suppressor signals from immunosuppressive macrophages. Importantly, upregulation of ERK1/2-mediated signaling pathways in T lymphocytes was linked to AR phosphorylation driven events. Shimizu et al. (140) concluded that AR mediates intracellular transmission of the suppressor signal of immunosuppressive macrophages toward downstream ERK1/2 pathways, possibly through its direct interaction with acceptor proteins.

Adding to the AR functional conundrum are data from mice devoid of AR (141). These AR null mice, otherwise normal from structural, biochemical, reproductive and physiological standpoint, display mild polyuria, and mild polydipsia (141), and moderately altered divalent cation levels (142). Nerve conduction velocity (NCV) is unaffected by the overexpression of AR; however, in a diabetic setting, in marked contrast to the fall in NCV in wild type mice, NCV is normal in the AR knockout mouse (141). Similarly, cardiac contractile function is unaffected by pharmacological inhibition of AR (143–145).

Though the physiological function of AR in normal cellular and organ physiology is unclear, the pathogenic role of AR as a key player mediating diabetic complications is well established. This review will focus on the pathogenic role of AR in diabetic cardiovascular complications.

## Diabetic Cardiac Ischemia and AR

The presence and activity of AR in cardiac myocytes of rats and rabbits has been demonstrated in several studies, e.g., (76, 143–147), and cardiac sorbitol and fructose tissue concentrations were shown to be significantly increased in diabetic rats compared to control rats (146). Studies have shown that diabetes and ischemia increase AR activity in hearts (76, 148) and that blockade of AR with ARI zopolrestat or sorbinil was found to improve cardiac glucose metabolism and to dramatically reduce acute ischemia-reperfusion-induced cardiac damage in diabetic rat hearts and in non-diabetic rat and rabbit hearts (76, 143–145).

**Figure 2:**
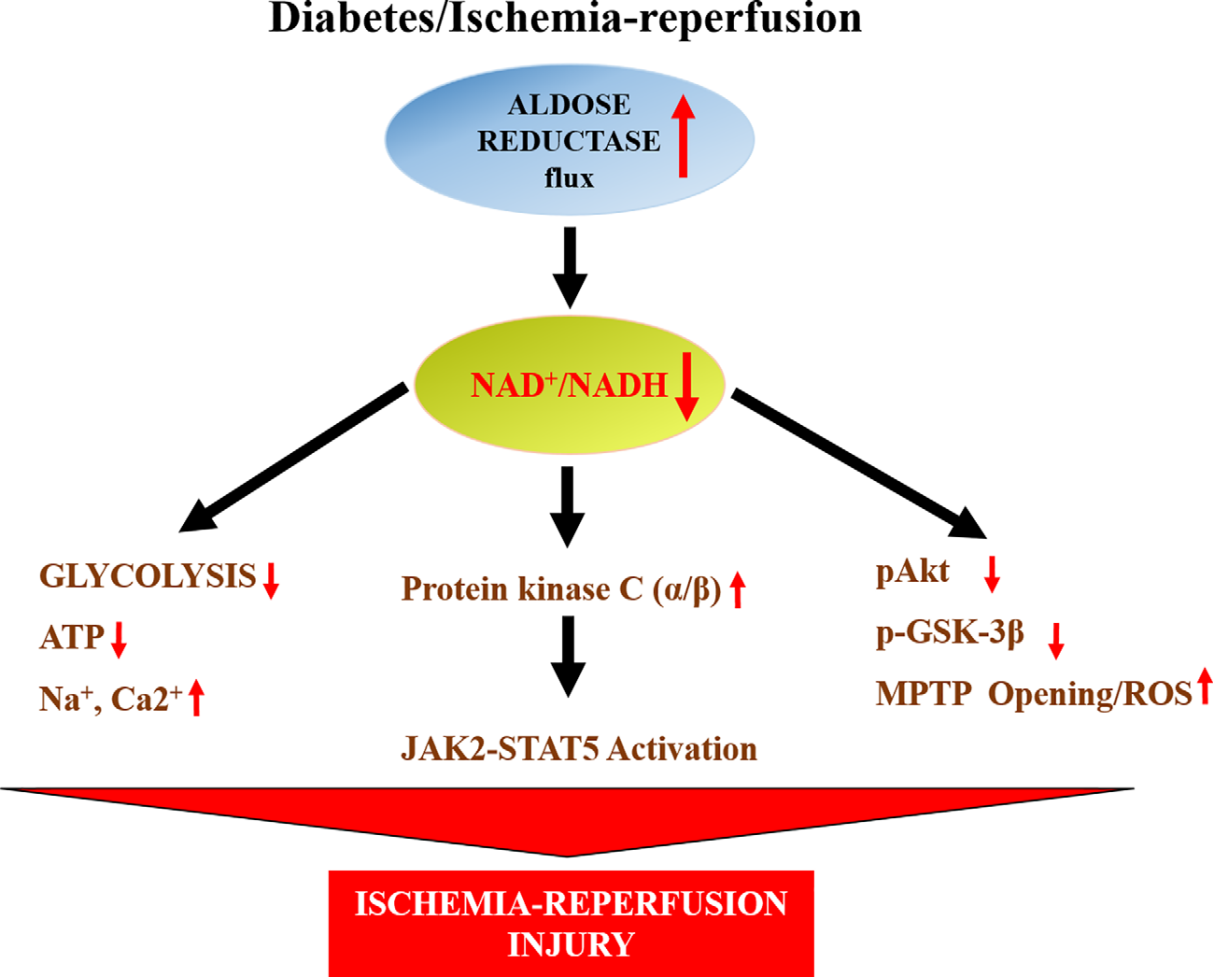
Scheme displays the impact of AR on changes in NAD^+^/NADH and consequent changes in glycolysis, mitochondrial properties and key signaling pathways leading to ischemic injury in hearts. ATP- adenosine triphosphate; JAK2- Janus activated kinase 2; MPTP-mitochondrial permeability transition pore, Akt- a serine/threonine-specific protein also known as Protein kinase B (PKB), pAkt- phosphorylated Akt, GSK3β-Glycogen synthase kinase 3 beta, STAT5- Signal transducer and activator of transcription 5.

Humans have much greater activity of AR than mice. For this reason, we used a transgenic mouse line in which human AR (hAR) was expressed *via* a histocompatibility gene promoter (149). These transgenic mice have tissue levels of AR activity comparable to those of humans (108). These hAR transgenic mice have been invaluable in recapitulating human diabetic cardiovascular disease. When subjected to ischemia/reperfusion (I/R), hearts from hAR transgenic mice exhibited greater injury, reduced ATP levels, and impaired functional recovery than wild-type mice (148). AR inhibitor zopolrestat attenuated I/R injury and improved functional recovery in these hAR transgenic mice (148). Studies in hAR transgenic mice addressing potential mechanisms revealed that opening the mitochondrial permeability transition pore (MPTP) (**Figure 2**) is linked to increased I/R injury (150). Increased generation of hydrogen peroxide, and reduced levels of antioxidant glutathione were key to MPTP opening in these hAR mice undergoing I/R (150). Attenuation of reactive oxygen species generation either by antioxidants or by ARIs reduced MPTP opening and reduced I/R injury in hAR transgenic mice hearts (150). Since MPTP opening is linked to phosphorylation of glycogen synthase kinase 3 β (GSK3β), subsequent studies in hAR mice and AR null mice, revealed that flux *via* AR reduces phosphorylation GSK3β *via* the Akt pathway in I/R hearts (151). These studies linked key signaling mechanisms by which AR impairs MPTP opening in I/R hearts.

Studies by Hwang et al. (152), in isolated perfused rat and mice hearts, revealed that ischemia drives JAK2 phosphorylation followed by STAT5 activation and that inhibition of AR or SDH blocked JAK2 and STAT5 activation (**Figure 2**). Furthermore, using pharmacological strategies they showed that the activation of JAK2-STAT5 pathway during ischemia in hAR mice was dependent on lowering of cytosolic NAD^+^/NADH and increased protein kinase C α/β activity. These data (152) (**Figure 2**), showed that AR mediates myocardial ischemic injury by modulating NAD^+^/NADH/protein kinase C α/β/JAK-STAT signaling.

To determine if AR actions in the heart are specifically in cardiomyocytes, we generated mice with cardiac specific expression of human AR (hAR) using the α-myosin heavy chain (MHC) promoter (153). Cardiomyocyte specific hAR transgenic expression did not alter cardiac function or glucose and fatty acid (FA) oxidation gene expression in young mice, whereas cardiac dysfunction was observed in older mice. Like the global hAR transgenic mice, these cardiac specific hAR mice also had greater infarct area and reduced functional recovery than non-transgenic littermates. In these studies, when the hAR transgene was crossed onto the PPAR alpha knockout background, hAR expressing mice had increased heart fructose content, cardiac fibrosis, reactive oxygen species (ROS), and apoptosis. These studies informed us that cardiomyocyte specific overexpression of hAR leads to cardiac dysfunction with aging and in the setting of reduced FA oxidation and increased glucose metabolism (153).

Studies addressing the short term and long term remodeling consequences of *in vivo* I/R injury model revealed that AR null mice was protected, in part, due to short term activation of the β-catenin pathway and subsequent increases in mesenchymal markers and fibrosis provoking genes (154). The increased activity of the β-catenin pathway and its downstream target genes in AR null mice was observed at early time points (48 h) of recovery after ligation of the descending coronary artery. At later time points of recovery (28 days), these changes in β-catenin activity were not observed in the AR null mice hearts. Thus, these data demonstrated that long term protection in AR null mice hearts was independent of β-catenin pathway.

*In vitro* and cellular studies described in the earlier sections of this review indicate that AR can detoxify aldehydes, such as 4-HNE, that accumulate during I/R. Studies in hAR expressing mice hearts have demonstrated increased injury and poor functional recovery after I/R (148, 155), along with increased oxidative stress. Furthermore, studies in AR-null mice hearts revealed reduced oxidative stress and reduced I/R injury (156). Similar findings linking increased AR activity and flux to increased oxidative stress has been demonstrated in rat hearts (157–161). It is possible that activation of aldehyde dehydrogenase 2 (ALDH2) reduces 4-HNE accumulation and protects hearts from I/R injury (162). Furthermore, it is possible that as shown in some studies, glutathione adduct of 4-HNE (GS-HNE) is converted by AR to its dihydroxynonane form (GS-DHN) and that inhibition of AR reduces GS-DHN and mitigates adverse signaling mechanisms driving inflammation and injury (163–165). In the context of diabetes, ALDH2 activity is known to be reduced in multiple tissue, including the heart (132, 133). Could the accumulation of 4-HNE observed in the diabetic hearts (especially during I/R) be due to lack of ALDH2? Comprehensive murine studies are warranted to establish the precise role of AR vs ALDH2 in modulating 4-HNE metabolism cascade in I/R hearts.

Like in I/R hearts, flux *via* AR is also increased in diabetic cardiomyopathy and heart failure. AR and SDH protein expression, activities and substrate flux were increased in hearts of Type 2 BBZDR diabetic rat hearts along with functional changes (166). AR expression was attenuated in pacing induced canine model of heart failure (167). Hearts tissue samples from patients with ischemic cardiomyopathy and diabetic cardiomyopathy exhibited elevated AR expression (168). These observations provide rationale for addressing the role of AR in mediating cardiac dysfunction and heart failure, both in diabetic and non-diabetic models.

In summary, the studies discussed establish AR as a key driver of functional and metabolic impairment in diabetic and ischemic hearts and that blockade of AR presents a therapeutic target for protection of these stressed hearts

## Atherosclerosis in Diabetes and AR

Patients with diabetes are at increased risk for CAD (5, 6). Gleissner et al. showed that AR is expressed in CD68^+^ cells (monocytes/macrophages) from human atherosclerotic plaques (108, 169), and that patients with diabetes had significantly greater CD68^+^AR^+^ macrophages in the plaques than patients without diabetes (170).

As discussed earlier, hAR transgenic mice (149) exhibit AR levels similar to those observed in humans. Previously, we reported that overexpression of hAR in LDL receptor knockout (*Ldlr^−/−^)* (171) and apolipoprotein E null (*Apoe^−/−^)*  (172) mice promoted atherosclerosis under hyperglycemic conditions and that pharmacological inhibition of AR reduced lesion size (172). Subsequent studies by us probed the mechanisms by which AR promoted atherosclerosis in hyperglycemic conditions.

Early events in atherosclerosis progression include endothelial dysfunction and upregulation of VCAM-1 (173). Vedantham et al. showed that, in both diabetic *Apoe^−/−^* mice and in human atherosclerotic carotid artery, AR is expressed in endothelial cells and this endothelial AR leads to endothelial dysfunction and increased expression of VCAM-1 and MMP-2 (148). Importantly, this study showed that AR inhibition improved endothelial function and was linked to attenuated VCAM-1 and MMP-2 expression (148) and that these findings were similar to those observed by blockade of RAGE in atherosclerotic *Apoe^−/−^* mice (174). Studies in cells linked AR to AGE and RAGE activation and consequent changes in intercellular adhesion molecule-1 and monocyte chemoattractant protein-1, migration, and monocyte adhesion (175) and that ARI or AR antisense oligonucleotides (176) blocked these changes, suggesting that, AR may promote progression of atherosclerotic plaques *via* AGE-RAGE axis. Pharmacological studies have shown that AR inhibitor treatments improves endothelium-dependent relaxation to acetylcholine of aortas from diabetic rabbits (176), diabetic rats (177), and galactosemic rats (177). Taken together, these findings establish a central role for AR pathway as a key mediator of impaired endothelium-dependent relaxation, endothelial dysfunction, cell adhesion, and inflammatory events in diabetic blood vessels.

**Figure 3:**
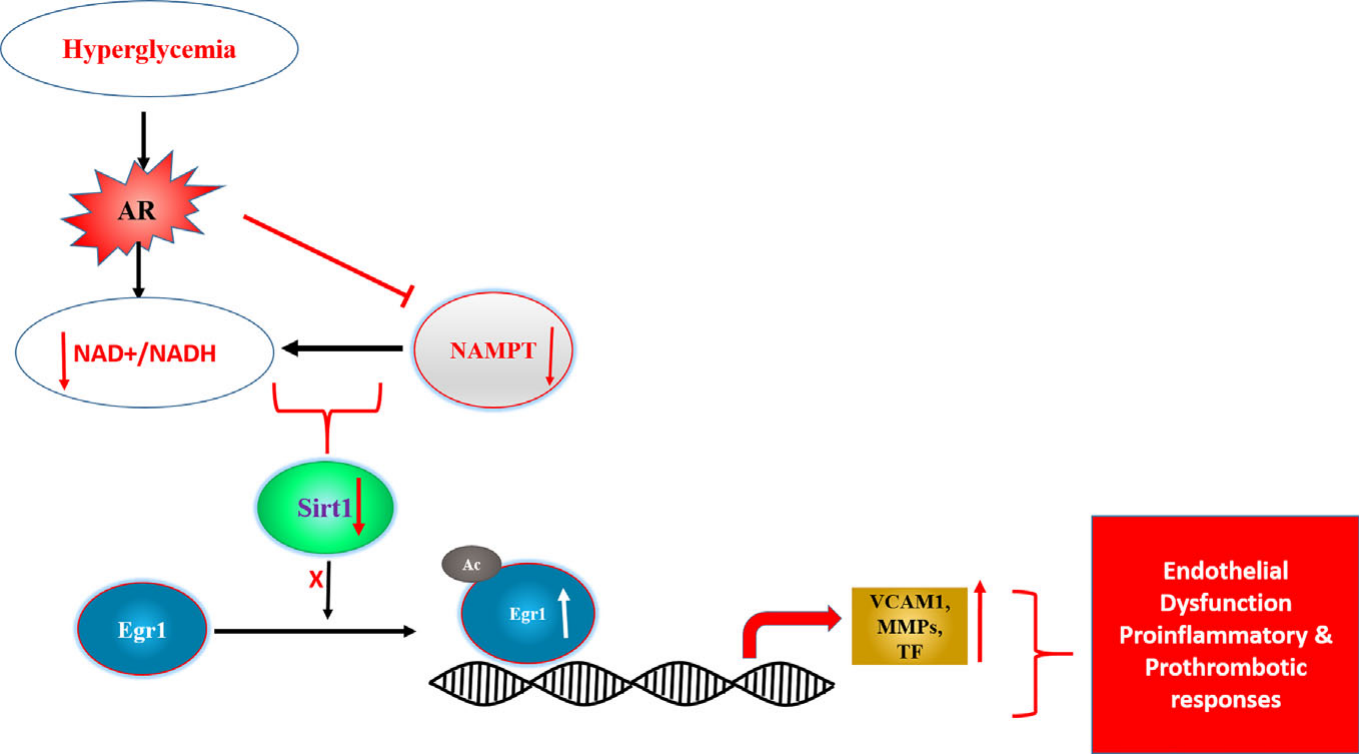
Scheme displaying AR driven changes in NAD^+^/NADH and SIRT1 activity as key driver of transcription factor Egr1 acetylation and consequent induction of proinflammatory and prothrombotic genes. [adapted from (169)]. Egr1- early growth response 1, SIRT1- NAD^+^ dependent Sirtuin1, Ac- acetylation, NAMPT-Nicotinamide phosphoribosyltransferase, VCAM1- vascular cell adhesion molecule 1, MMPs- matrix metalloproteinases, TF-tissue factor.

Mechanisms probing cellular and *in vivo* studies to address link between AR and inflammation, revealed that flux *via* AR impaired drives inflammatory gene changes *via* Egr-1. Specifically, changes in AR activity and flux reduces NAD^+^ levels triggering reduced activity of NAD^+^-dependent deacetylase Sirt-1 and consequent acetylation and prolonged expression of Egr-1 in hyperglycemic conditions (**Figure 3**) (178). These data established a novel AR-SIRT1-EGR1 mechanism by which glucose may lead to proinflammatory and prothrombotic responses in diabetic atherosclerosis.

While the studies in hAR overexpressing mice revealed that AR promotes atherosclerosis progression in diabetes, an unexpected finding of increased early lesion size was observed in diabetic *Apoe^−/−^*mice devoid of AR (179). In this study lesion size positively correlated with 4-HNE in mice devoid of AR, which the authors postulated was likely to reduced metabolism of toxic aldehydes. Given the potential impact of ALDH-2 in detoxifying 4-HNE (162) and recent studies by Singh et al. showing that macrophages from AR null mice exhibit higher basal and lipopolysaccharide stimulated phagocytic activity  (180), additional studies are warranted to understand the mechanisms in play when AR is deleted in *Apoe^−/−^*mice. Human AR expression does appear to recapitulate human diabetic atherosclerosis more closely in *Ldlr^−/−^* and *Apoe^−/−^* mice models, suggesting that, AR deletion may have unintended consequences, including compensatory regulation influencing vascular properties.

Diabetic patients demonstrate impaired atherosclerosis regression and persistent absolute risk level of a cardiovascular event following lipid lowering drugs compared to nondiabetic patients. Murine studies, in atherosclerosis regression models, attributed the impairment to hyperglycemia-induced monocytosis and recruitment of these macrophages to plaques (181). Yuan et al. (182), using Type 1 diabetic Akita mice with and without hAR overexpression and aortic transplantation model, addressed the role of AR in impaired atherosclerosis regression in diabetes. In the surgical model of atherosclerosis regression, the donor aortic arch containing the preformed atherosclerotic plaques are transplanted into a recipient mice that are kept on normal chow diet. Yuan et al. transplanted donor aorta into the following recipient mice; either *Ldlr^−/−^*, non-diabetic wild type, Akita, hAR transgenic, or Akita/hAR mouse. In the recipient Type 1 diabetic mice, hyperglycemia significantly impaired the decrease in percent of CD68^+^ lesion area, even after hyperlipidemia was attenuated. The combination of Akita with overexpression of hAR significantly increased the percent of lesion macrophage content in the plaques, suggesting continued atherosclerosis progression. Plaque CD68^+^ cells from the *Akita^+/-^*/hAR mice demonstrated increased oxidant stress as measured by DHE fluorescence. They also exhibited higher expression of genes linked to pro-inflammation and reduced expression of anti-inflammatory genes. This study demonstrated that hAR expression amplifies impaired atherosclerosis regression in Type 1 diabetic mice. Taken together, the atherosclerosis progression and regression studies in diabetes demonstrate a key pathogenic role for AR and that interventions to block AR may be beneficial in diabetic atherosclerosis.

## AR and platelets in diabetes

Platelet abnormalities, one of the hallmarks of diabetes, contributes to the pathogenesis of atherosclerosis and thrombosis. Studies by Tang et al. (183), in human platelets, demonstrated that AR plays a key role in mediating thromboxane release, increased cell surface thromboxane receptor expression, and enhanced platelet activity in human platelets treated with hyperglycemic conditions and/or collagen. Importantly, they linked these changes to increased oxidative stress and the activation of PLCγ2, PKCβII, PKCδ, and p38α MAPK (183). Furthermore, studies in diabetic subjects and humanized AR transgenic mice rendered diabetic with STZ revealed that hyperglycemia driven AR activation and subsequent increases in oxidative stress leads to increased p53 phosphorylation, followed by mitochondrial dysfunction, damage, and rupture of platelets by sequestration of the antiapoptotic protein Bcl-x_L_ (184). Taken together, these human and animal studies established that AR is key mediator of abnormal platelet activity in diabetes, thus adding to the multiple processes that contribute to the pathogenesis of diabetic cardiovascular complications.

## AR and Vascular Injury

Diabetes is known to cause increased restenosis after angioplasty. AR plays a central role in smooth muscle cell (SMC) proliferation caused by balloon injury in animal models of restenosis. Studies have shown that AR inhibition prevents SMC growth in in animal models of restenosis (185–190). Studies in cells and tissue demonstrated that high glucose flux *via* the AR pathway leads to diacylglycerol accumulation and consequent protein kinase C activation (186). In addition, AR was shown to modulate hyperglycemia and TNF-α driven increases in the extracellular signal–related kinase/mitogen-activated protein kinase and phosphatidylinositol 3-kinase, (187), as well as activation of nuclear factor κB (188), and G1/S-phase proteins E2F-1, cdks, and cyclins (191). These signaling changes lead to upregulation of SMC chemotaxis, vascular inflammation, and cell adhesion. AR inhibition attenuated the above signaling events and arrested proliferation and migration of SMCs. Findings from these cellular and animal studies provide a compelling rationale for testing AR inhibitors for safety and efficacy in diabetic patients undergoing angioplasty and at risk for restenosis (185–190).

## AR Inhibitors and Properties

AR inhibitors (ARIs) have been extensively reviewed in the literature, e.g., (53, 83, 192). At this time, epalrestat is the only ARI that is being used, in Japan, India, and China, to treat patients with diabetic neuropathy (98, 192). X−ray crystallographic studies of ARIs revealed that they bind in the active site of AR. Most ARIs that have been tested in human trials belong to the chemical classes of spirohydantions or carboxylic acids (192, 193). ARIs of the carboxylic acid class are quite selective for AR vs. aldehyde reductase (192, 194). One efficacy challenge of the carboxylic acid class of ARIs is that they are highly protein bound *in vivo*. Hydantoin class of ARIs inhibit both aldehyde and AR with comparable efficacies (192, 195), thus likely to cause to off target effects. Another strategic approach that is under active consideration is the design and use of inhibitors to preferentially inhibit glucose reduction while preserving the detoxifying ability of AR toward toxic aldehydes (196, 197).

The number of patents filed over the last 5 years demonstrates that, after decades of uncertainty, scientific interest in AR and its inhibitors has resurged. During the last 5 years, a number of synthetic compounds have been designed and patented as AR inhibitors, mainly belonging to the carboxylic-type class. Inspired by the well-known inhibitor zopolrestat, Mylari and co-workers designed a novel class of carboxylic acid inhibitors and its water-soluble formulations (198–201) to overcome some of the limitations of this class of ARIs. Shendelman recently patented two novel series of phthalazino and pyrazinopyridazino derivatives (202). Although these compounds are closely related to ones described by Mylari and coworkers, in their heterocyclic portion, the novel derivatives possess a boronic residue that replaces the carboxylic acid moiety. This gives the ARI field a rather new chemical approach for developing active inhibitors. These newly developed ARIs are actively being tested for its efficacy in animal and human diabetic cardiovascular disorders.

## Clinical Applications of ARIs in Humans

Initial studies on AR inhibition attenuating injury and improving functional recovery after I/R in both diabetic and nondiabetic hearts generated considerable interest toward testing these molecules for diabetic heart disease (143, 144, 148, 203, 204), and for development of new ARIs (193, 194). In clinical studies, AR inhibitor, zopolrestat, treated diabetic subjects displayed increased left ventricular ejection fraction (LVEF), cardiac output, left ventricle stroke volume and exercise LVEF (205) whereas, placebo-treated diabetic subjects exhibited decreased exercise cardiac output, stroke volume and end diastolic volume (205). Didangelos et al. showed that AR inhibition beneficially altered heart rate variability in patients with severe or moderate diabetic autonomic neuropathy (206). These promising studies in human subjects with established diabetic complications paved the way for the development and use of new ARIs, such as AT-001, currently in clinical trials. Multicenter, randomized, placebo-controlled, 2-part study to evaluate the safety and efficacy of AT-001, a novel AR inhibitor, in adult patients with diabetic cardiomyopathy at high risk of progression to overt heart failure, is currently in progress (NCT04083339). AT-001 treatment for 28 days was shown to reduce blood levels of sorbitol and N-terminal pro-B-type natriuretic peptide levels in diabetic patients (207). In addition to its role in mediating cardiac dysfunction and injury, preclinical studies have shown that AR exacerbates lung inflammation (204). Taken together these studies formed the basis for the current testing of ARI in COVID19 patients [see review by Kadosh et al. (208)]. Currently, AR inhibitor, AT-001 is undergoing trials to assess safety and efficacy in reducing inflammation and cardiac injury in COVID-19 diabetic patients with heart disease (NCT04365699). Taken together, these findings from animal and human studies strongly suggest that AR promotes diabetic cardiovascular complications. Current large randomized multicenter human trials using the newly developed potent ARIs are likely to establish its therapeutic potential in diabetic cardiovascular complications.

## Conclusions

The DCCT, UKPDS, and prior ARI studies have indicated that relatively long clinical trials with focused recruitment strategies and end points will be needed to demonstrate efficacy on microvascular and macrovascular complications of diabetes. Focus on prevention or slowing of cardiovascular disease progression in diabetic patients should be the primary goal, not rapid reversal of disease endpoints. Despite developmental setbacks over the last two decades, preclinical and clinical evidence linking progression of diabetic cardiovascular complications and elevated flux *via* AR unambiguously confirm pathogenic role for AR in mediating diabetic complications. Importantly, data on genetic polymorphisms *of ALD2* from around the globe indicate an association between “high AR” alleles and diabetic complications. The preclinical and clinical data reviewed above indicate that inhibiting AR could play a key role in our therapeutic strategy to prevent/arrest progression of diabetic cardiovascular complications. Recent development of potent AR inhibitors and new formulations has set the stage for successful clinical testing of these molecules in patients with diabetic cardiovascular complications.

## Author Contributions

SJ, MS, and RR wrote the first draft of the manuscript and completed all of the editing. GY and RR prepared figures. SJ, MS, GY, AS, and RR provided critical comments on the manuscript and edited the manuscript. All authors contributed to the article and approved the submitted version.

## Funding

AS and RR are supported, in part, by funds from the Diabetes Research Program, NYU Grossman School of Medicine and grants from the U.S. Public Health Service (P01HL143697, R01HL132516, and R01DK109675 (to AS and RR). SJ was supported by a training grant for medical students [T35 DK007421, RR (PI)]. RR is a consultant for Applied Therapeutics and receives funding from them for ARI studies.

## Conflict of Interest

The authors declare that the research was conducted in the absence of any commercial or financial relationships that could be construed as a potential conflict of interest.
